# Ten Years' Research on a Cardiovascular Tonic: A Comprehensive Approach—From Quality Control and Mechanisms of Action to Clinical Trial

**DOI:** 10.1155/2013/319703

**Published:** 2013-11-12

**Authors:** Ping-Chung Leung, Chi-Man Koon, Clara Bik-San Lau, Ping Chook, William King-Fai Cheng, Kwok-Pui Fung, Timothy Chi-Yui Kwok, Kam-Sang Woo

**Affiliations:** ^1^Institute of Chinese Medicine, The Chinese University of Hong Kong, Hong Kong; ^2^State Key Laboratory of Phytochemistry and Plant Resources in West China, The Chinese University of Hong Kong, Hong Kong; ^3^School of Medical Sciences, The Chinese University of Hong Kong, Hong Kong; ^4^Department of Medicine and Therapeutics, Faculty of Medicine, The Chinese University of Hong Kong, Hong Kong; ^5^School of Life Sciences, The Chinese University of Hong Kong, Hong Kong; ^6^School of Life Sciences, Biochemistry Programme, The Chinese University of Hong Kong, Hong Kong

## Abstract

*Objective*. Mortality arising from cardiovascular pathologies remains one of the highest. Maintenance of cardiovascular health therefore remains a universal concern. Interventional therapies and medications have made impressive advances, but preventive measures would be of the same importance. *Method*. Ten years' search for a simple herbal formula has resulted in a two-herb combination, consisting of *Salviae Miltiorrhizae Radix et Rhizoma* and *Puerariae Lobatae Radix*. The formula has been studied extensively on cardiovascular biological platforms and then put on three clinical trials. *Results*. In the laboratory, the formula was found to have the biological effects of anti-inflammation, anti-oxidation, anti-foam cell formation on vascular endothelium, and vasodilation. Clinical trials using ultrasonic carotid intima thickness as a surrogate marker showed very significant benefits. No significant adverse effects were encountered. *Conclusion*. It is therefore recommended that the herbal formula could be used as an adjuvant therapy in cardiac patients under treatment or as a preventive agent among the susceptible.

## 1. Introduction

The aging population commonly suffers from deteriorating cardiovascular health. Indeed, mortalities related to cardiac failure and cerebral vascular accidents have remained the major causes of death. Although remedial measures are available to maintain cardiovascular well-being, from therapeutic measures to highly sophisticated revascularisation skills, adverse drug effects and recurrences of obstructions are still inevitable [1]. The search for agents that protect the cardiovascular system on a broad base, without being too specific, is a logical attempt [2].

Some herbs in the Chinese medicine have been widely used for the promotion of “circulatory strength,” which in modern terms should mean cardiovascular health. A wide variety of proprietor herbal preparations are available in market and people in the Chinese communities have been using them either in combination with pharmaceuticals like aspirin and statins or as prophylactic agents for blood cholesterol control and/or vascular integrity [3, 4]. We intend to choose, among the many popular herbs traditionally used for cardiovascular problems, the least number to form a simple combined formula to be used as an effective cardiovascular protective tonic. The mechanisms of action need to be explored properly before the formula would be put on an evidence-based clinical trial. 

### 1.1. The Two-Herb Formula—Danshen and Gegen (D&G)

 Among the many medicinal herbs *Salviae Miltiorrhizae Radix et Rhizoma *(Danshen) stands out as the most frequently used one. Its clinical values and vascular protective effects have such strong historical background that users take it for granted for its efficacy claims [5]. Given a full respect to the philosophy of clinical treatment in the Chinese medicine, we need to identify one more herb to form a combined formula and to gain enhanced effects or synergies.

A deceased respectable herbal expert and clinician, Shi Jin-mo (1882–1968), was well known for his expertise on selecting twin combinations of herbs in the formation of simple, synergistic formulae. Shi advocated the use of *Salviae Miltiorrhizae Radix et Rhizoma* (Danshen) together with *Puerariae Lobatae Radix* (Gegen) for the promotion of a good circulation [6]. Many proprietary manufactures have since made more complicated formulae, based on Shi's recommendation of Danshen and Gegen (D&G).

Danshen and Gegen together, therefore, constitute a simple herbal formula (D&G) favourable for further study on biological platforms to prove its efficacy. 

### 1.2. Quality Control and Chemical Fingerprint

Danshen was purchased from Sichuan province and Gegen from Guangdong province of China. Both places are noted for the best quality supply of the respectable herb. Large batches, estimated to be sufficient for both the laboratory and later clinical trials, were acquired to ensure uniformity.

The raw herbs were morphologically authenticated by a botanist and chemically using the thin layer chromatography in accordance with the Chinese Pharmacopoeia 2005. Small quantities of the two raw herbs were deposited as voucher specimens in the sample bank of the Institute of Chinese Medicine, The Chinese University of Hong Kong, with voucher specimen numbers of 2008-3166a and 2008-3167a for Danshen and Gegen, respectively. 

They were then washed, cut into small pieces, and weighed in the ratio of 7 : 3. The herbs were soaked with 10-fold of water (v/w) for 1.5 hr, followed by extraction at 100°C for 1 hr. Two subsequent extractions were carried out with 10-fold of water (v/w) for another 1 hr and 0.5 hr. The extracts were combined and concentrated under reduced pressure to give dry D&G powdered extract.

Accurately weighed 0.5 g sample was sonicated with 20 mL methanol for 30 min at 40°C. The solution was filtered and evaporated to dryness. The residue was re-dissolved in 5 mL methanol and filtrated through a 0.45 *μ*m syringe filter. The final extract was further diluted 5 times for LC-DAD-MS analysis.

Chemical analysis was done and recorded using LC-DAD-MS instrumentation with set conditions. An Agilent 1100 Series LC/MSD Trap VL (Agilent Technologies, USA) coupled with a photodiode array detector was used. The mass spectra were acquired using ion trap instrument with an ESI source. The ESI source was operated at a sheath gas (N_2_) flow of 30 psi, auxiliary gas (N_2_) flow of 10.0 L/min, an ion spray voltage of 3.5 kV, and a capillary temperature of 340°C. For chromatographic separation, the column consisted of a Thermo ODS hypersil reserved-phase column (5 *μ*m, 250 mm × 4.6 mm) and a Thermo ODS hypersil guard column (5 *μ*m, 10 mm × 4.6 mm). The sample injection volume was 10 *μ*L. The detection wavelength was set at 254 and 280 nm, the flow rate was 0.8 mL/min and the column temperature was maintained at 20°C. The mobile phase consisted of 0.8% acetic acid (A) and acetonitrile (B) and operated at gradient separation. The initial condition was A-B (98 : 2, v/v) and remains unchanged for 10 min. Over the next 50 min, the percentage of mobile-phase B increased linearly to 30%. Then the percentage of mobile-phase B increased linearly to 50% on the next 20 min [7–9].

The chemical fingerprint of D&G was thence established and registered. 

## 2. Methods

### 2.1. Biological Studies

If D&G was cardiovascular protective, one could expect it to be anti-inflammatory, anti-oxidative, and might be anti-coagulant. These biological activities were serially verified on *in vitro* cell line models. The tests include the following. 

#### 2.1.1. Anti-Inflammatory and Anti-Oxidative Tests

- Inhibition of LPS—induced nitric oxide production [10].
- Inhibition of iNOS, COX_2_, and NF*κ*B protein expression using Western blot [11].
- Inhibition of inflammatory cytokines using ELIZA [12].
- Inhibition of NF*κ*B translocation using electrophoretic motility shift assay (EMSA) [11].
- Inhibition of iNOS and COX_2_ inflammatory cytokines gene expressions using real-time PCR [12].
- Inhibition of foam cell formation using macrophages (RAW 264.7) acetylated low-density lipoprotein uptake [13].

#### 2.1.2. Vascular Protection Tests

- Effect of D&G on blood pressure, using spontaneous hypertensive rats (SHRs) [12].
- Effect of D&G on vasodilation using *ex vivo* aortic ring of rats [5].
- Effect of D&G on balloon injury-induced neointimal media thickness [14, 15].
- Effect of D&G on cerebral blood flow using the middle cerebral artery occlusion rat model to evaluate neurological deficit, brain infarct, and anti-oxidative effects on brain tissues [16–19].
- Effect of D&G on myocardium [20–22].

#### 2.1.3. Tests on Cardiac Effects (Zebrafish Embryo)

- D&G effects on heart rate [20].
- D&G effects on acetylcholine and on *β* adrenergic activities [17].
- D&G effects on cardiac toxicity.

#### 2.1.4. Functional Genomic Studies

- Using rat cardiac myoblast cell line H9c2 exposed to different doses of D&G to check cell proliferation and cell cycles, and using cDNA microarray analysis to identify the 5 categories of genes, namely, cardiovascular, apoptosis, cell proliferation, cytokine and inflammation, and anti-oxidants.
- Variations were induced through hypoxia treatment and pretreatment with D&G.
- Tissue specific gene expression pattern, protein expression profiles, and signaling pathways involved were also studied [23, 24].

#### 2.1.5. Herb-Drug Interaction: Whether D&G Interfere with Systemic Anti-Coagulant (Warfarin)

It is important to understand whether D&G might enhance or lower the anti-coagulant effects of standard, maintenance therapies that many patients are receiving.

- D&G was given together with warfarin to rats, to check tail bleeding time, prothrombin time, and platelet agglutination.
- Basic pharmacodynamics studies also included interaction with aspirin and diclofenac sodium.
- Basic bioavailability of D&G using their marker compounds was also studied [25, 26].

### 2.2. Clinical Trials

The three clinical trials were designed as randomized, double-blind, placebo-controlled clinical studies.

*Trial 1*. The clinical trial was aimed to evaluate the efficacy and safety of *Salvia miltiorrhiza* (Danshen) and *Pueraria lobata* (Gegen) in secondary prevention. One hundred (100) eligible coronary patients were randomized to take 6 capsules of the D&G preparation (3 g) or 6 capsules of placebo capsules daily, in a double-blind and parallel fashion for 24 weeks. Brachial flow-mediated dilation (FMD) and carotid intima-media thickness (IMT) were measured using ultrasound technology.

*Trial 2*. Atherosclerosis commonly occurs in patients with hypertension. We hypothesized that Danshen and Gegen (D&G) have beneficial effects on the atherogenesis of high-risk hypertensive subjects. 90 patients with essential hypertension (SBP 160/90 mmHg before treatment) were studied. All subjects were randomized to receive either oral D&G capsules 1 g/day, D&G capsules 2 g/day, or placebos, in a double-blind parallel fashion for 12 months. Brachial flow-mediated dilation (endothelium-dependent dilation, FMD) and carotid intima-media thickness (IMT) were measured using ultrasound technology. 

*Trial 3*. This clinical study was designed to demonstrate the safety and effectiveness of D&G in the prevention of atherosclerosis in postmenopausal women with early hypercholesterolemia. 165 postmenopausal women were randomized to take the D&G preparation (2 capsules) or placebo capsules (2 capsules) daily, in a double-blind and parallel fashion for 12 months. Carotid intima-media thickness (IMT) was measured using ultrasound technology. The lipid profile was also tested. 

## 3. Results

### 3.1. Biological Studies

#### 3.1.1. Anti-Inflammatory Effects

The direct anti-inflammatory effects and the indirect effects through anti-oxidative mechanisms, foam cell inhibition, and so forth were all positively demonstrated [13]. 

#### 3.1.2. Vascular Protection

The anti-hypertensive effects of D&G on spontaneous hypertensive rats (SHRs) were clearly shown. The endothelium-independent vasodilatation effects and the nitric oxide related mechanisms were shown in *ex vivo* isolated rat aorta rings model [5, 12, 16–19].

The balloon injury model demonstrated the inhibitory effects of D&G on the deposition of atheromatous plugs [14, 15].

#### 3.1.3. Cardiac Effects

D&G reduced acetylcholinesterase activity in zebrafish embryonic hearts. D&G induced bradycardia in zebrafish embryos through the regulation of muscarinic and *β* adrenergic pathways [17, 20]. 

#### 3.1.4. Functional Genomic Studies

To understand the molecular mechanism of the cardio protection effect of D&G, the functional specific cDNA microarray was used to study the expression profile of genes related to cardic disease biomarker, apoptosis, cell cycle and proliferation, cytokine and inflammation, and anti-oxidants. A homemade rat cDNA microarray containing 100 genes was fabricated to study gene expression profiles of H9c2 cells upon 50 *μ*g/mL of D&G treatment for 24 hr. After data analysis, it was found that 14 and 11 relevant genes were either upregulated or downregulated by D&G treatment, respectively [23, 24]. 

Our study demonstrated that D&G could promote the expression of apolipoprotein D (*Apod*), lecithin cholesterol acyltransferase (*Lcat*), and intercellular adhesion molecule 1 (*Icam1*), which are well-known cardiac biomarkers. D&G could also upregulate the expression of inducible nitric oxide synthase (*iNos*) and downregulate the selection expression. 

The results suggested that the D&G might exert its protective effects on myocardial cells by regulating NO and the selection expression. 

The study demonstrated that both IRS-1 and AKT were activated in the D&G-treated myocardial cells. It is well known that AKT could promote cell survival and oppose apoptosis by a variety of routes. The study suggested that several routes might be involved in the cardiac protection effect of D&G. For example, the induction of the phosphorylation of I kappa B leads to the activation of the transcription factor nuclear factor kappa B (NF-*κ*B) to suppress apoptosis. The promotion of the expression of nitric oxide synthase, which can catabolize L-arginine to NO, triggers many physiological actions in the cardiovascular system. 

The study showed that D&G could negatively regulate the expression of tumor necrosis factor-*α* (TNF-*α*) at both gene and protein expression levels. Tissue specific genes, protein expression patterns, and signaling pathways, involved in SHR aorta and heart, treated with D&G were found. 

Furthermore, to examine the treatment group- and tissue-specific gene expression profiling induced by D&G, the differentially expressional genes from different groups of tissues were studied, and the results resembled observations described as above.

#### 3.1.5. Pharmacokinetic Study

With regard to the pharmacokinetic study of the identified markers after oral administration of D&G, the whole pharmacokinetic profiles of the important chemicals like danshensu, puerarin, and daidzein could be obtained, whereas salvianolic acid B, protocatechuic aldehyde, and daidzin could not be detected possibly because of extremely low quantity. Moreover, the assay for the simultaneous determination of R-warfarin and S-warfarin and their metabolites in rat plasma had been developed. The results showed that coadministration of D&G with warfarin/aspirin would result in significant pharmacokinetic and pharmacodynamic (prothrombin time and bleeding time were increased) interactions in the rat. More in-depth studies would be required in future before the wide clinical uses of D&G.

### 3.2. Clinical Trials****

*Trial 1*. The baseline characteristics were similar between the 2 groups. After 6 months' treatment, there were no significant changes in blood pressures, blood hematological and biochemical profiles, folate, and homocysteine levels in both groups when compared with the baseline but a mild decrease in low density lipoprotein cholesterol in both groups ((2.6 ± 0.7) mmol/L versus (2.7 ± 0.9) mmol/L, *P* < 0.05; (2.5 ± 0.7) mmol/L versus (2.8 ± 0.8) mmol/L, *P* < 0.05). The brachial FMD was improved after treatment in the D&G group (5.9% ± 1.0% versus 5.3% ± 1.2%, *P* < 0.001), and it was less improved in control group (5.5% ± 1.0% versus 5.3% ± 1.1%, *P* < 0.05). Improvement in carotid IMT was observed in the D&G group only, and it has significance ((0.96 ± 0.32) mm versus (0.98 ± 0.30) mm, *P* < 0.05). After the open-label D&G treatment for 6 more months, further improvement in both brachial FMD and carotid IMT was observed in the D&G group, and they had significance (5.91% ± 0.95% versus 5.35% ± 1.21%, *P* < 0.05; (0.964 ± 0.316) mm versus (0.979 ± 0.303) mm, *P* < 0.05). Eight adverse events were reported: 2 in the D&G group; 6 in the control group, among which, 2 patients required treatment termination.

*The Conclusion Made*. Danshen and Gegen adjunctive treatment for patients with coronary arterial diseases was well tolerated and effective in improving vascular function and structure [27, 28]. 

*Trial 2*. To evaluate the potential of D&G for primary atherosclerosis prevention in high-risk hypertensive patients, 90 patients (74.4% male) with hypertension associated with left ventricular hypertrophy (63.3%), diabetes mellitus (62.5%), and renal insufficiency (30%) were randomized to receive D&G herbal capsules (2 gm/day) or (1 gm/day) or identical placebo capsules in double-blind and parallel fashion for 12 months on top of their anti-hypertensive treatments. Flow-mediated dilation (endothelium-dependent dilation, FMD) and nitroglycerin-induced dilation (endothelium-independent dilation, NTG) of brachial artery and carotid intima-media thickness (surrogate atherosclerosis marker, IMT) were measured by high resolution B-mode ultrasound. 

Results showed that their mean age was 55 ± 8 years. After 12 months and compared with the baseline, there were no significant changes in blood pressure, heart rate, blood cholesterol (TC), haematological, glucose (HBAIc), and creatinine profiles in both placebo and D&G groups. FMD and IMT but not NTG improved significantly after D&G (*P* < 0.001) and not after placebo treatment. No significant difference in FMD and IMT changes in the 2 D&G groups with different dosages was seen. The studied herbal drugs were well tolerated in both groups, with no significant adverse events reported.

*The Conclusion Made*. Danshen and Gegen adjunctive treatment was well tolerated and significantly improved the atherogenic process in high-risk hypertensive patients. There was potential in the primary prevention of atherosclerosis [29–32].

*Trials 3*. A population based sample of 165 postmenopausal women aged 47 to 65 was included in the trial. Only women who experienced menopause for more than 12 months were recruited.

Results showed that the baseline characteristics were comparable between the 2 groups. After 12 months, there were no significant changes in blood pressures and general biochemical profiles in both groups. However, there was a remarkable decrease in serum low density lipoprotein (LDL) cholesterol (−6.92%) and total cholesterol (TC) (−5.85%) from the baseline in the D&G group, when compared with placebo group (−3.21% and −3.42%). The carotid intima-media thickness (IMT) decreased 1.52% from the baseline in the D&G group (*P* < 0.004), and the decrease was only 1.13% for the placebo treatment group (*P* = 0.009) after a 12-month treatment. Twelve adverse events were reported: 6 in the placebo group and 6 in the D&G group; no one of them was significant.

*The Conclusions Made*. Postmenopausal women with early hypercholesterolemia tolerated D&G well. The D&G improved the carotid intima and lowered LDL and total cholesterol. D&G therefore may be recommended for the prevention of atherosclerosis in postmenopausal women with hypercholesterolemia [33]. 

## 4. Discussion

The comprehensive approach to the creation of an evidence based simple herbal formula for cardiovascular health has taken ten years to reach the present state of maturity. In the laboratory, through a variety of *in vitro* platforms, we have demonstrated the multiple biological activities of D&G, namely, anti-inflammation, anti-oxidation, and anti-form cell formation on the vascular endothelium. The different mechanistic channels leading to these favourable cardiovascular protective events have also been demonstrated in the extensive cytokine studies. Through a variety of animal studies, D&G has been demonstrated to provide control on hypertension, atherosclerosis, and vasodilatation. D&G appears to be both cardiac protective and vascular protective. 

The traditional Chinese medicine is characterized by its complexity and holism in both diagnostic and therapeutic approaches. DNA microarray technology could be a powerful tool to study the functional genomics of the traditional Chinese medicine. It might be useful in the identification and characterization of the active components of the complex mixtures to provide significant information for understanding the efficacy of the herbs from the genomic point of view in a systematic way and to hunt for candidate disease genes or characterization of tissue-specific genes. In this study, the genomic and proteomic signatures of D&G treated samples either *in vitro* or *in vivo* were investigated using cDNA microarray and iTRAQ labeled LC/MS/MS techniques, respectively, which provide better understanding of the mechanism of action of Danshen-Gegen. Our future challenge is to integrate the information to give a more complete picture of the interaction between the herbal formula and the living organisms.

We look forward to the more sophisticated microarray studies which might lead to more definitive identification of sub-fractionations within the D&G extract to give more targeted preparations. 

With regard to the bioavailability study of the D&G oral administration, the whole pharmacokinetic profiles of the important chemicals like danshensu, puerarin, and daidzein could be obtained, whereas salvianolic acid B, protocatechuic aldehyde, and daidzin could not be detected or were found under the limit of quantification. Moreover, the assay for the simultaneous determination of R-warfarin and S-warfarin and their metabolites in the rat plasma was developed. The results showed that the coadministration of D&G with warfarin/aspirin would result in significant pharmacokinetic and pharmacodynamic changes which would require more studies.

We have conducted three randomized controled clinical trials using the same surrogate markers on different target populations. Firstly, we chose the coronary type II patients who were at high risks. D&G served them well. Next, we chose the less risky patients (those with hypertension and/or diabetes mellitus). D&G also gave good results. Lastly, we recruited postmenopausal women with borderline increase of cholesterol. D&G helped maintain the low cholesterol level. At this stage, we are sure that D&G did not give rise to serious adverse effects. It is a safe preparation and deserves further in-depth studies.

## 5. Conclusion

This safe preparation has been developed from very popular edible medicinal herbs. The formula has been advocated by a respectable Chinese medicine physician. The current preparation with a modified ratio has shown multiple mechanisms of biological activities which are beneficial to the cardiovascular system. Now that we have reliable means to maintain the quality of the two herbs through careful assessment of their chemical and biological profiles, we could confidently recommend that D&G is a safe and an effective choice for cardiovascular protection. Its further development could follow the direction of a proprietary medicine for a proper hospital and specialist use or as a specific health supplement, targeting towards cardiovascular health. 
